# A Heterozygous LMF1 Gene Mutation (c.1523C>T), Combined With an LPL Gene Mutation (c.590G>A), Aggravates the Clinical Symptoms in Hypertriglyceridemia

**DOI:** 10.3389/fgene.2022.814295

**Published:** 2022-03-16

**Authors:** Danxia Guo, Yingchun Zheng, Zhongzhi Gan, Yingying Guo, Sijie Jiang, Fang Yang, Fu Xiong, Hua Zheng

**Affiliations:** ^1^ Department of Cardiovascular Medicine, Nanfang Hospital, Southern Medical University, Guangzhou, China; ^2^ Department of Medical Genetics, School of Basic Medical Sciences, Southern Medical University, Guangzhou, China; ^3^ Department of Fetal Medicine and Prenatal Diagnosis, Zhujiang Hospital, Southern Medical University, Guangzhou, China

**Keywords:** hypertriglyceridemia, lipase maturation factor 1, lipoprotein lipase, missense mutation, heterozygous mutation

## Abstract

Hypertriglyceridemia is an important contributor to atherosclerotic cardiovascular disease (ASCVD) and acute pancreatitis. Familial hypertriglyceridemia is often caused by mutations in genes involved in triglyceride metabolism. Here, we investigated the disease-causing gene mutations in a Chinese family with hypertriglyceridemia and assessed the functional significance *in vitro*. Whole-exome sequencing (WES) was performed revealing that the severe hypertriglyceridemic proband carried a missense mutation (c.590G > A) in exon 5 of the *LPL* gene, as well as a missense mutation (c.1523C > T) in exon 10 of the *LMF1* gene. Conservation analysis by Polyphen-2 showed that the 508 locus in the LMF1 protein and 197 locus in the LPL protein were highly conserved between different species. I-TASSER analysis indicated that the *LMF1* c.1523C > T mutation and the *LPL* c.590G > A mutation changed the tertiary structure of the protein. A decrease in mRNA and protein expression was observed in 293T cells transfected with plasmids carrying the *LMF1* c.1523C > T mutation. Subcellular localization showed that both wild-type (WT) and mutant LMF1 protein were localized at the cell cytoplasm. In the cell medium and cell lysates, these *LMF1* and *LPL* gene mutations both caused a decreased LPL mass. Moreover, the combination of *LMF1* and *LPL* gene mutations significantly decreased LPL levels compared to their individual effects on the LPL concentration. Both the clinical and *in vitro* data suggest that severe hypertriglyceridemia was of digenic origin caused by *LMF1* and *LPL* mutation double heterozygosity in this patient.

## Introduction

Associated with the occurrence and development of acute pancreatitis and atherosclerotic cardiovascular disease (Hegele et al., 2014), hypertriglyceridemia (HTG) is a common disease characterized by triglyceride levels higher than 150 mg/dl (1.7 mmol/L). Notably, when triglyceride levels exceed 1,000 mg/dl, the incidence of acute pancreatitis will significantly increase to 5% (Scherer et al., 2014). To the best of our knowledge, HTG is related to environmental and genetic factors, and is induced by common conditions. These include pregnancy, hypothyroidism, chronic kidney disease, and metabolic syndrome (Pearce, 2004; Goldberg and Hegele, 2012; Catapano et al., 2016). Primary severe HTG has both monogenic and polygenic determinants. Similarly, a range of monogenic and polygenic variants can lead to either the severe or mild-to-moderate primary HTG. Familial HTG is often caused by loss of function mutations in the five main genes, namely lipoprotein lipase (*LPL*), apolipoprotein C2 (*APOC2*), apolipoproteinA5 (*APOA5*), glycosylphosphatidylinositol-anchored high-density lipoprotein binding protein 1 (*GPIHBP1*), or lipase maturation factor 1 (*LMF1*), that regulate TG-rich lipoprotein lipolysis.

LPL is the key enzyme in systemic lipid metabolism that mediates intravascular hydrolysis of triglycerides into chylomicrons and very-low-density lipoprotein (VLDL). As a lipase chaperone located in the endoplasmic reticulum, LMF1 is required for LPL folding and/or dimerization. Homozygous mutations in a locus called cld were found to cause severe HTG for the first time in the mouse model in previous studies, and heterozygous mice had normal TG levels (Péterfy et al., 2007). Peterfy et al. (Péterfy et al., 2007) identify the cld gene and name it *Lmf1*, and also identify a human individual with combined LPL deficiency who was homozygous a nonsense *LMF1* mutation. In humans, while a few common or rare *LMF1* variants have been reported in HTG cases, no clear evidence of functional analyses exists. Only three homozygous nonsense *LMF1* mutations (p.Tyr439*, p.Tyr460*, and p.Trp464*) have been identified as being causative of hyperchylomicronemia. The p.Ser137Leu missense variant in the *LMF1* gene was shown to be causative of severe hypertriglyceridemia through functional analysis (Péterfy et al., 2018).

In this study, we investigated a Chinese family spanning four generations that has been diagnosed with HTG. The clinical phenotypes and the functions of the pathogenic mutations were analyzed in this family so as to study the pathogenesis of HTG.

## Materials and Methods

### Patients

This study was approved by the Nanfang Hospital Ethics Committee, an affiliate of the Southern Medical University (Guangdong, China). Written informed consent was obtained from all participants. The proband (II-4), a 43-year-old male, was admitted to the Nanfang Hospital’s Department of Cardiology with repeated fasting serum triglyceride (TG) concentration measurements exceeding 20 mmol/L. Plasma lipid profile analysis and other routine examinations of the proband and his family members, which included his father, mother, brother, daughter and son, were performed in Nanfang Hospital.

### Mutation Screening

Blood samples obtained from the proband were sent to the Nanfang Hospital Precision Medicine Center for Whole-exome sequencing (WES) of genomic DNA. Illumina HiSeq platform was used for WES. The criteria established and revised by the American College of Medical Genetics and Genomics (Richards et al., 2015) was used to classified the variants. Two hypertriglyceridemia associated genes mutations, within exon 10 of the *LMF1* gene and exon 5 of the *LPL* gene identified by WES, were verified using Sanger sequencing. Standard phenol/chloroform extraction was performed to extract genomic DNA from the peripheral blood acquired from the proband and his family members (Ⅰ1, Ⅰ2, Ⅱ4, Ⅱ2, Ⅲ4, and Ⅲ3). The PCR primers that were designed using Primer-BLAST (https://www.ncbi.nlm.nih.gov/tools/primer-blast) were as follows: *LMF1*: Exon-10 forward primer: 5′-CCG​TCT​CAG​CCA​CCA​GAA​AA-3′, Exon-10 reverse primer: 5′-CAC​GGC​TGG​TTT​GGT​TTG​AG-3'; and *LPL*: Exon-5 forward primer: 5′-CCA​GCC​ATC​CTG​AGT​GGA​AA-3′, Exon-5 reverse primer: 5′-GGCTCTAAGGTGGTCATGCT-3'.The PCR products were then analyzed by agarose gel electrophoresis and submitted to Invitrogen (Shanghai, China) for Sanger sequencing.

### Bioinformatics

For WES, BaseSpace BWA Enrichment App was used to perform read alignment and variant calling. The sequence was aligned with BWA Genome Alignment Software. Variant calling was performed with GATK using the human reference sequence GRCh37. Variant annotation was performed by ANNOVAR software. Available genomic databases (dbSNP, 1,000 Genomes Project, Exome Variant Server, Exome Aggregation Consortium and a local Paris Descartes Bioinformatics platform database) were used to filter exome variants and exclude variants with a frequency >1%.

The three-dimensional (3D) protein structures of wild-type and mutant LMF1 and LPL proteins were predicted using I-TASSER (Iterative Threading ASSEmbly Refinement, http://zhanglab.ccmb.med.umich.edu/I-TASSER/). Amino acid sequences were submitted to the online I-TASSER software, which then provided the predicted 3D protein structure. The conservation of LMF1 and LPL proteins was analyzed by Polyphen-2 (Polymorphism Phenotyping v2, http://genetics.bwh.harvard.edu/pph2/) to predict the harmfulness of mutations.

### Plasmid Constructs and Mutagenesis

Total RNA was extracted from peripheral blood using Trizol reagent (Invitrogen, California, United State). The HiScript II 1st Strand cDNA Synthesis Kit (Vazyme, Jiangsu, China) was used for the synthesis of first-strand cDNA according to the manufacturer’s instructions. The coding sequence (CDS) regions in *LMF1* gene were amplified using primers containing BglII and SalI restriction sites, while the CDS regions in *LPL* gene were amplified using primers containing NheI and BamHI-HF restriction sites. The primers were as follow: *LMF1*: forward primer: 5′-CGA​GAT​CTA​TGC​GCC​CTG​ACA​GCC​CAA-3′, reverse primer: 5′- ACG​CGT​CGA​CAA​GAG​GGG​CCC​GGG​CAG​AG-3′; and *LPL*: forward primer: 5′-CTA​GCT​AGC​ATG​GAG​AGC​AAA​GCC​CTG​C-3′, reverse primer: 5′-CGG​GAT​CCG​CCT​GAC​TTC​TTA​TTC​AGA​GAC​TTG-3′. The WT coding region of the *LMF1* gene was then cloned into the BglII and SalI sites of the pEGFP-N1 vector (GENEWIZ, New Jersey, United States) and the WT coding region of the *LPL* gene were cloned into the NheI and BamHI-HF sites of the pcDNA3.1-cFLAG plasmid (Life Technology, Thermo Fisher Scientific, Waltham, Massachusetts, United States). Moreover, the mutation plasmid were generated by PCR-based site-directed mutagenesis. The primers for this were as follows: *LPL*: forward primer: 5′-CAG​AAG​CCC​CGA​GTC​ATC​TTT​CTC​CTG​ATG-3′, reverse primer: 5′- CAT​CAG​GAG​AAA​GAT​GAC​TCG​GGG​CTT​CTG-3′; and *LMF1*: forward primer: 5′- TCG​CGG​GCA​GGC​CCC​TGC​CCA​GGT​GGG​TCC-3′, reverse primer: 5′- GGA​CCC​ACC​TGG​GCA​GGG​GCC​TGC​CCG​CGA-3′. Recombinant plasmids were purified using the Plasmid Miniprep Kit (Axygen, New York, United States) and sequenced to exclude the presence of random mutations.

### Cellular Localization

Human embryonic kidney 293T cells (HEK293T cells) were used for functional analysis of *LMF1* variants. HEK293T cells were cultured in Dulbecco’s Modified Eagle Medium (DMEM; Gibco, New York, United States) supplemented with 10% fetal bovine serum (Gibco) at 37°C and 5% CO_2_.

The subcellular location of LMF1 proteins was detected by immunological fluorescence assays. The recombinant plasmids containing wild-type or mutant *LMF1* genes were transfected into HEK293T cells using Lipofectamine 2000 (Invitrogen) according to the manufacturer’s instructions. After 36 h, the successfully transfected HEK293Tcell were fixed for 30 min with 4% paraformaldehyde (Sigma-Aldrich, St. Louis, MO, United States) after washing three times with PBS (Sigma-Aldrich). Following discarding of the paraformaldehyde, the samples were again washed three times with PBS. Cells were then incubated in 0.1% Triton X-100 (Thermo Fisher Scientific, Massachusetts, United States) to increase the permeability of the cytomembrane. Nuclei were stained with 4′, 6-diamidino-2-phenylindole (DAPI; Sigma). The stained cells were viewed using a confocal fluorescence microscope (LSM 880; Carl Zeiss AG, Jena, Germany).

### RNA Analysis

HEK293T cells were transfected with 1 μg of the recombinant plasmids containing wild-type or mutant genes using Lipofectamine™ 2000 transfection reagent (Invitrogen). After 36 h of transfection, total RNA was extracted from the cell lysates using Trizol reagent (Invitrogen) and reverse transcribed into cDNA using the PrimeScript™ RT reagent Kit (Takara, Dalian, China). Quantitative real-time PCR was performed to compare the relative mRNA levels between lysates of HEK293T cells transfected with wild-type and mutant genes using the 2 × RealStar Green Fast Mixture (GenStar, Beijing, China). The GAPDH gene was chosen as the reference gene. The primers were as follows: *LMF1*: forward primer: 5′-CGT​AAC​AAC​TCC​GCC​CCA​TT-3′, reverse primer: 5′-TCCGAGTACCCA GTCTTCCG-3'; and GAPDH: forward primer: 5′-GTG​AAG​GTC​GGA​GTC​AAC​G-3′, reverse primer: 5′-TGA​GGT​CAA​TGA​AGG​GGT​C-3'. RNA analyses were repeated three times, and there was three groups of sample in each time (*n* = 9).

### Western Blot Analysis

HEK293T cells transfected with the recombinant plasmids containing wild-type or mutant genes were collected and washed with cold PBS (Sigma-Aldrich). Cell lysis buffer (Beyotime Biotechnology, Shanghai, China) supplemented with 1% phenylmethanesulfonyl fluoride (PMSF, Beyotime Biotechnology) was used to lyse cells and prevent protein degradation. The concentration of cell lysates was determined using the Pierce™ Rapid Gold BCA Protein Assay Kit (Thermo Fisher Scientific) after removing cell debris by centrifugation. The obtained protein samples were separated by sodiumdodecyl sulfate-polyacrylamide gel electrophoresis (SDS-PAGE) and transferred to a polyvinylidene fluoride (PVDF) membrane (Millipore, Massachusetts, United States). The membranes were blocked with 5% nonfat milk for 60 min at room temperature, and then incubated with GFP-tagged mouse monoclonal antibody (Ray Antibody Biotech, Beijing, China) or anti-GAPDH mouse monoclonal antibody (Sigma Aldrich) overnight at 4°C. After washing with Tris Buffered Saline-Tween 20 (TBST) three times, the membranes were incubated with goat anti-mouse IgG (Sigma-Aldrich) at room temperature for 2 h. Protein expression was detected by SuperSignal West Pico ECL (Thermo Fisher Scientific) and a digital chemiluminescence system (Tanon Science& Technology, Shanghai, China). ImageJ software was used to quantify the intensities of the protein bands. LMF1 expression was normalized against GAPDH levels. Western blot analyses were repeated three times, and there was three groups of sample in each time (*n* = 9).

### ELISA Analysis

HEK293T cells were transfected with a total of 3 μg of the recombinant plasmids containing wild-type or mutant *LPL* and *LMF1* genes (*LPL*:*LMF1* of 2:1) using Lipofectamine™ 2000 transfection reagent (Invitrogen). Thirty hours post-transfection, 1 ml of complete medium with10 U heparin was added into the cells cultured in 6-well plates, and the supernatants were collected after centrifugation. The cell lysates were obtained by freeze-thaw cycles. The samples were then analyzed using the human lipoprotein lipase (LPL) ELISA KIT (LunChangShuo Biotechnology, Xiamen, China) in accordance with the manufacturer’s instructions. ELISA analyses were repeated three times, and there was two groups of sample in each time (*n* = 6).

### Statistical Analyses

Statistical analyses were performed using GraphPad Prism software. Data were presented as the mean ± standard deviation (SD). The independent samples t-test was used for determining the statistical significance of the differences between the two groups. A *p*-value < 0.05 was considered to be statistically significant. Significance was further indicated using asterisks, where *p < 0.05, **p < 0.01, ***p < 0.001, and ****p < 0.0001.

## Results

### Clinical Features

**FIGURE 1:**
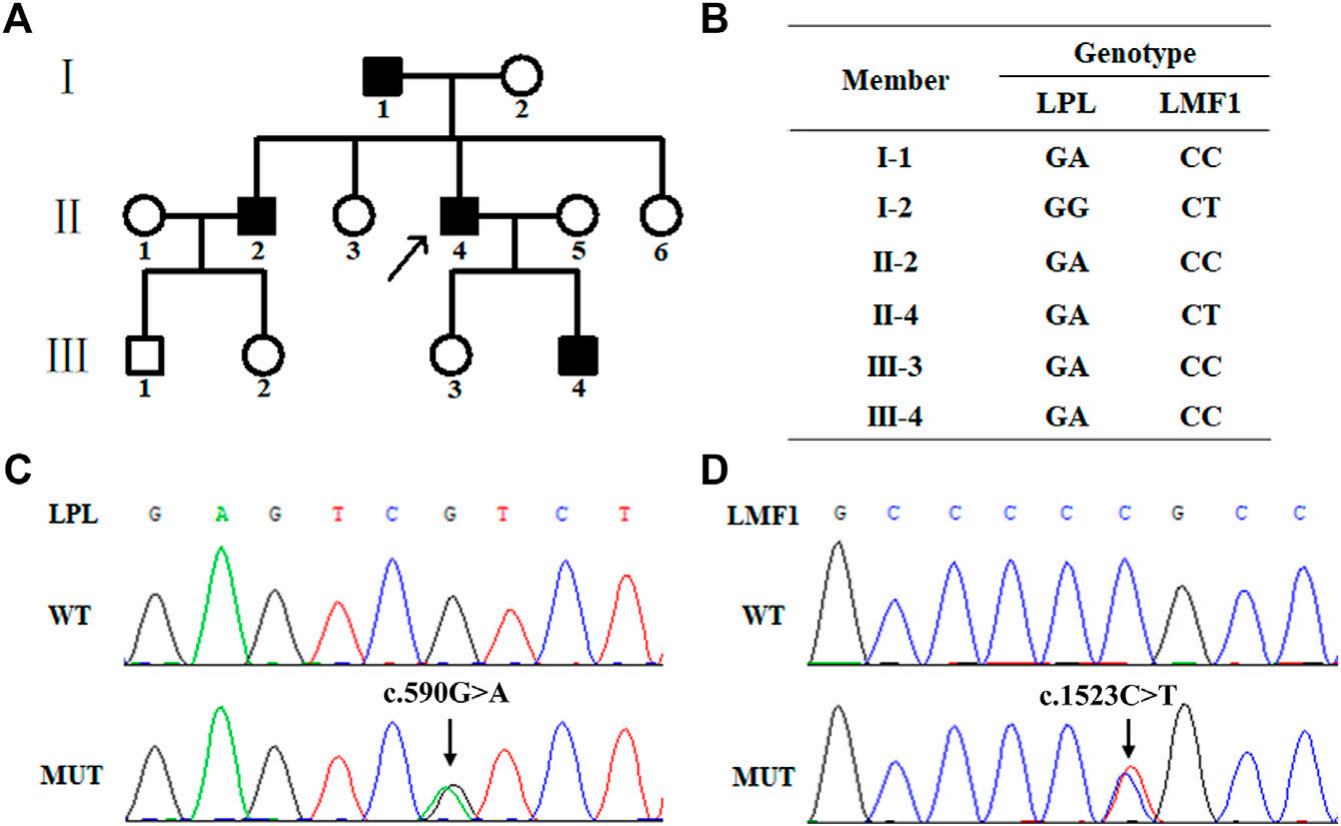
Family pedigree and mutation screening of samples from a family with hypertriglyceridemia. **(A)**. Family pedigree. The arrow indicates the proband. **(B)**. The genotype of six family members. The proband (Π4), І1, ш4 and ш3 carry the heterozygous missense mutation (c.1523C > T) in the *LPL* gene. The proband (Π4) and his mother (І2) carry heterozygous missense mutation (c.590G > A) in the *LMF1* gene. **(C)**. Sanger sequencing results of the heterozygous mutation (c.1523C > T) in the *LPL* gene. **(D)**. Sanger sequencing results of the heterozygous mutation (c.590G > A) in the *LMF1* gene.

The proband (II-4), a 43-year-old male, was admitted to the Nanfang hospital Department of Cardiology with repeated measurements of fasting serum TG concentration exceeding 20 mmol/L. Neither acute pancreatitis nor eruptive xanthoma occurred. The proband had no history of chronic kidney disease, diabetes mellitus, or hypothyroidism. On the occasion of his admission, the proband presented with serum TG levels of 25.19 mmol/L. After the onset of lipid lowering drug therapy (fenofibrate 0.2 g qd, and ezetimibe 10 mg qd) in the first day of admission, the serum TG levels of the proband decreased steadily to 8.15 mmol/L. The plasma lipid profile of his family members is shown in Table 1. The proband, I-1, II-2, and III-4 are HTG patients charactered by plasma triglyceride levels of ＞1.7 mmol/L. The proband’s mother and daughter are healthy (Figure 1A)

**TABLE 1:** plasma triglyceride levels in the proband and his relatives.

	Genotype		TG(mmol/L)	TC(mmol/L)	LDL-C(mmol/L)	VLDL-C(mmol/L)	HLDL-C(mmol/L)	nHLDL-C(mmol/L)	Sd(mmol/L)
	LPL	LMF1							
Ⅰ1	GA	CC	5.06	4.99	2.26	1.87	0.86	4.13	1.32
Ⅰ2	GG	CT	1.70	8.94	6.74	0.46	1.74	7.20	3.10
Ⅱ2	GA	CC	12.92	5.06	1.39	—	1.15	—	—
Ⅱ4	GA	CT	25.19	9.20	1.10	7.62	0.48	8.72	—
Ⅲ3	GA	CC	0.83	5.62	3.71	0.40	1.51	4.11	0.99
Ⅲ4	GA	CC	5.78	5.48	2.87	1.94	0.67	4.81	1.35

TG, triglyceride; TC, total cholesterol; LDL-C, Low-Density Lipoprotein Cholesterol; VLDL-C, Very-Low-Density Lipoprotein Cholesterol; HLDL-C, High-density lipoprotein cholesterol; n HLDL-C, non-High-density lipoprotein cholesterol; sd, small dense Low-Density Lipoprotein.

### Genetic Analysis

WES was performed using genomic DNA from peripheral blood obtained from the proband to identify the possible pathogenic mutation. The proband carried a missense mutation (c.1523C > T) in the *LMF1* gene (GenBank accession no. NM_022773 exon10, p.Pro508Leu, rs372213215) and a missense mutation (c.590G > A) in the *LPL* gene (GenBank accession no. NM_000237 exon5, p.Arg197His, rs372668179). These genes regulate TG-rich lipoprotein lipolysis. These two variants were classified as uncertain clinical significance, PM2, PP2 and PP3 for *LPL* c.590G > A, and PM2, PP3 for *LMF1* c.1523C > T. The genotype of the proband and his five family members is shown in Figure 1B. The proband (Ⅱ4), his father (Ⅰ1), his brother (Ⅱ2), his son (Ⅲ4), and his daughter (Ⅲ3) carried the c.1523C > T missense mutation, while the proband (Ⅱ4) and his mother (Ⅰ2) carried the c.590G > A missense mutation (Figures 1C,D).

### Functional Analysis of LMF1 and LPL

**FIGURE 2:**
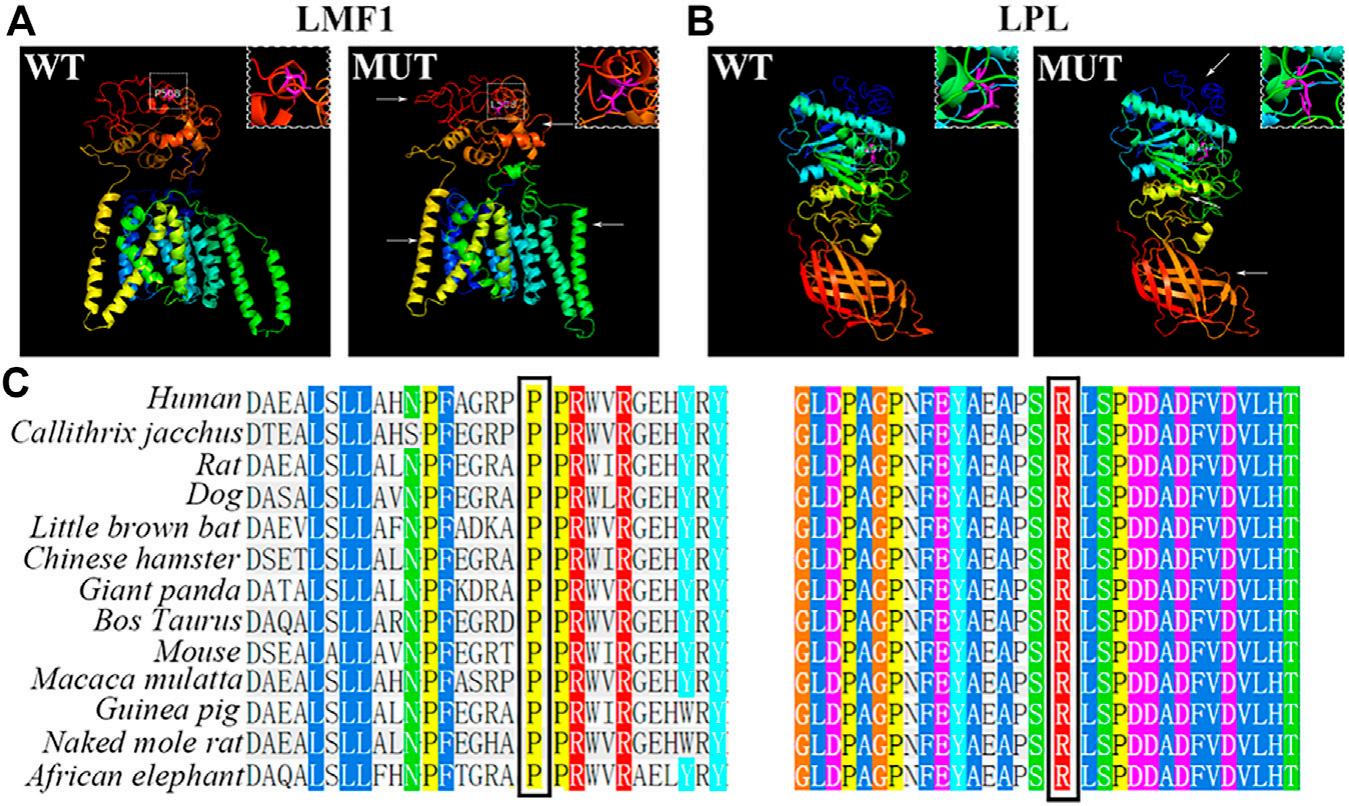
Bioinformatics analysis of the mutation. **(A)**. Prediction of wild-type (left) and mutant (right) protein structures of LMF1 by I-TASSER. **(B)**. I-TASSER prediction of wild-type (left) and mutant (right) protein structures of LPL. **(C)**. Sequence alignment of mutant amino acids on LMF1 (P508) or LPL (R197) across different species. White box: the mutated position; Arrows: changes compared to the wild-type structure; Magenta: mutant amino acids.

**FIGURE 3:**
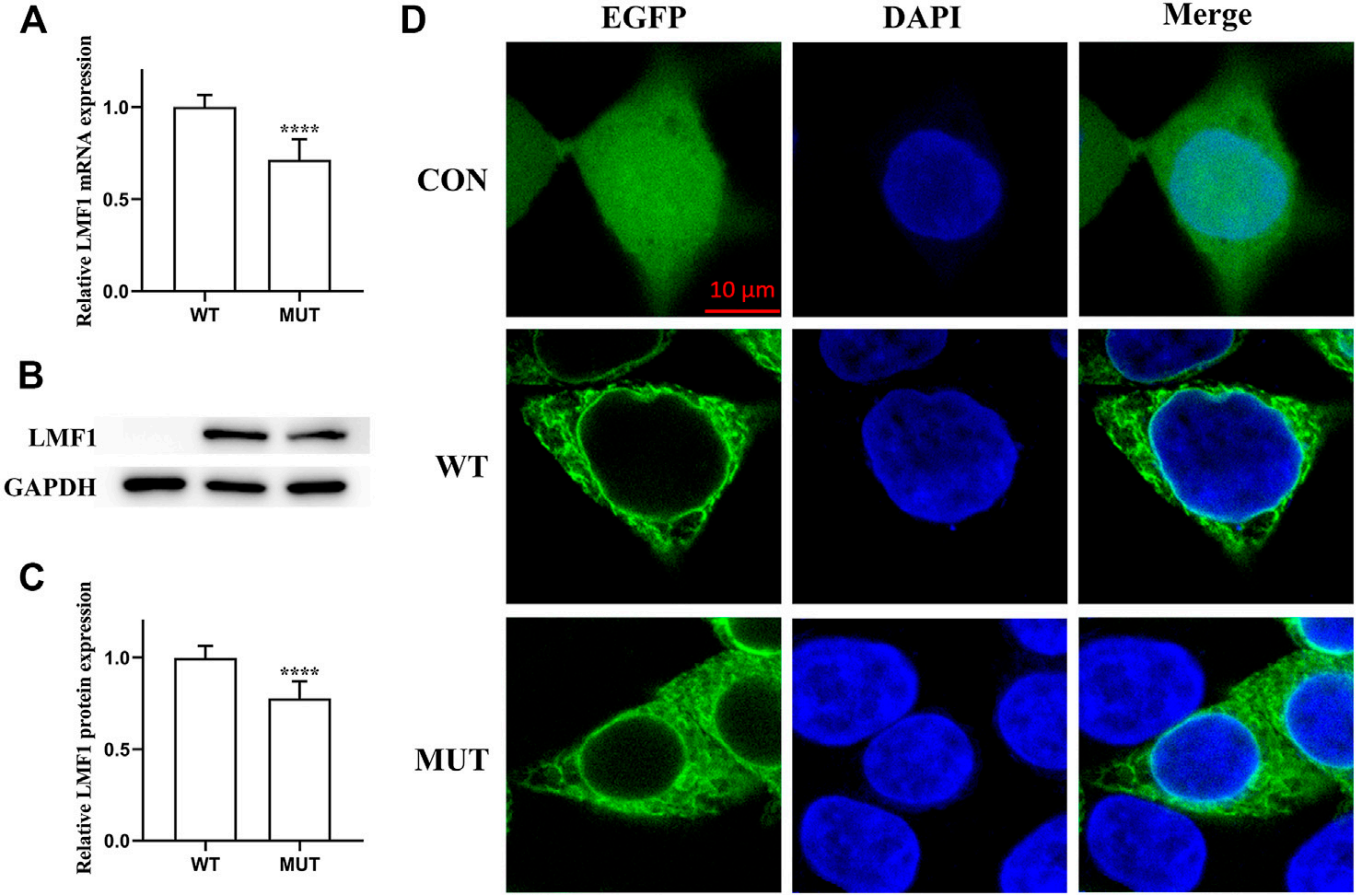
Functional analysis of the LMF1 mutant protein. **(A)**. mRNA expression levels of wild-type and mutant LMF1 genes in HEK293T cells. There was a significant difference in the mRNA level of the wild-type and the mutant genes (****p < 0.0001). **(B)**. Western blotting of LMF1 expression. **(C)**. Protein expression levels of wild-type and mutant LMF1 genes in HEK293T cells. The mutant LMF1 protein expression was lower than that of the wild-type proteins in HEK293T cells (****p < 0.0001). **(D)**. Subcellular localization of wild-type and mutant LMF1 in HEK293T cells.

The conservation analysis of the *LMF1* and *LPL* gene loci, P508 and R197, respectively, showed that the two mutations were located in evolutionary conserved regions. As shown in Figure 2C, the proline amino acid at LMF1 position 508, and arginine amino acid at LPL position 197, are consistent across different species. Additionally, I-TASSER indicated that the *LMF1* c.1523C > T mutation and the *LPL* c.590G > A mutation both altered the tertiary structure of the protein (Figures 2A,B). The helix changed into coil in the mutant position. Changes in other position of the structure were also found. The cellular localization of the LMF1 protein was analyzed in HEK293T cells transfected with wild-type and mutant constructs. The proteins were both localized in the cytoplasm and no differences were observed in the subcellular localization between the mutant and wild-type proteins (Figure 3D). Moreover, we found significant differences in the levels of LMF1 mRNA and protein expression using wild-type and mutant constructs in HEK293T cells. As shown in Figures 3A–C, mutant LMF1 mRNA and protein expression were both lower than that of the wild-type in HEK293T cells.

### *LMF1* and *LPL* Double Mutants Significantly Decreased LPL Levels

**FIGURE 4:**
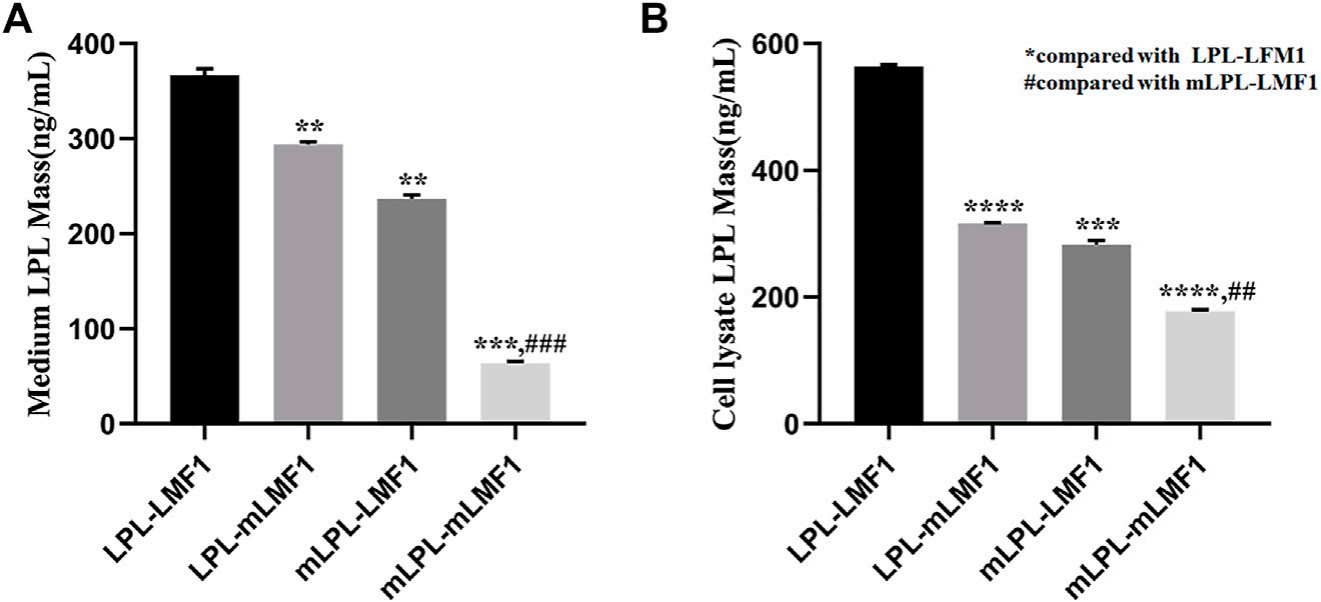
LMF1 and LPL double mutants significantly decreased LPL levels. **(A)**. The *LMF1* and *LPL* gene mutation both caused decrease of LPL levels in the cell medium. The combination of *LMF1* and *LPL* gene mutation significantly decreased LPL mass in the cell medium (*compared to LPL-LMF1, #compared to mLPL-LMF1; ****p < 0.0001, **p < 0.01). **(B)**. The *LMF1* and *LPL* gene mutation caused decrease of LPL mass in the cell lysate. The combination of *LMF1* and *LPL* gene mutation significantly decreased LPL mass in the cell lysate (*compared to LPL-LMF1, #compared to mLPL-LMF1; ##*p* < 0.01, ***p < 0.001, ****p < 0.0001).

To evaluate the effect of the *LMF1* mutations, the level of LPL in HEK293T cells transfected with plasmids carrying wild-type, mutant *LPL* and *LMF1* genes were measured. As shown in Figures 4A,B, these *LMF1* and *LPL* mutations both caused a decreased LPL level in the cell medium and cell lysates compared to the wild-type LPL concentrations. Moreover, *LMF1* and *LPL* double mutants (mLPL-mLMF1) significantly decreased LPL level in comparison to the decrease observed for the *LMF1* (c.1523C > T) or *LPL* (c.590G > A) mutation homozygote (LPL-mLMF1 or mLPL-LMF1).

## Discussion

In this study, a heterozygous *LMF1* missense mutation (c.1523C > T) and a heterozygous *LPL* missense mutation (c.590G > A) were identified in the proband who presented with severe HTG using WES. The mutations were confirmed in the proband and his family members by Sanger sequencing. Within the family, there are four *LPL* c.590G > A heterozygous carriers, one *LMF1* c.1523C > T heterozygous carriers, and one double *LPL/LFM1* heterozygote. Except the proband’s daughter, the *LPL* c.590G > A heterozygous carriers were diagnosed with HTG. The proband who carried double *LPL/LFM1* heterozygote had extremely high TG levels. The heterozygous mutation was first reported in a male Caucasian patient with severe HTG by Wright in 2008 (Wright et al., 2008). Surendran and Rabacchi subsequently also identified this mutation in HTG patients (Surendran et al., 2012; Rabacchi et al., 2015). These studies merely mention the *LPL*-associated c.590G > A mutation found in HTG patients. In this study, it was demonstrated that the c.590G > A mutation in the *LPL* gene could result in a decreased LPL mass in the cell medium and the cell lysates. Compared clinical characteristics of patients carried *LPL* c.590G > A mutation, age and TG level were in a big different, also the history of pancreatitis were different. The proband’s daughter, who carries the c.590G > A mutation, presented with normal TG levels. Nevertheless, the regulation of triglyceride is a complex process. Since her LPL levels were not measured, there is a possibility that she carried a different mutation, which could also increase the LPL activity. It was reported that the p.Thr143Met mutation, which could increase the activity of LPL, was identified in a patient with Familial hyperchylomicronemia (Plengpanich et al., 2020).

The c. 1523G > A missense mutation in the *LMF1* gene has not previously been linked to HTG. Moreover, the allele frequency of the *LMF1* c. 1523C > T variant in the ExAC and gnomAD databases for the East Asian population was 0.0001 and 5.831e-05, respectively. In short, the *LMF1* c. 1523C > T variant is extremely rare in populations when the allele frequency less than 0.1%.

Deficiency of LMF1 was identified in a cld mouse model characterized with a progressive increase in triglycerides that resulted in death 2–3 days after birth (Péterfy et al., 2007). Thus far, most mutations in the *LMF1* gene are heterozygous mutations found in HTG patients, but there is a lack of empirical evidence to support their pathogenicity. Only three rare nonsense variants, p.Tyr439Ter, p.Tyr460Ter, and p.Trp464Ter, have been reported on both alleles of the *LMF1* gene in HTG cases (Péterfy et al., 2007; Cefalù et al., 2009; Cefalù et al., 2017). The first two homozygous *LMF1* truncating mutations have previously been experimentally demonstrated to impact LMF1 activity. To date, p.Ser137Leu was identified and reported as the first missense mutation affecting LMF1 function (Péterfy et al., 2018). This variant severely diminishes the expression of mutant LMF1 and dramatically reduces the specific activity of LMF1. The c.1523C > T mutation in our study also decreased the expression of LMF1 mRNA and protein. *LMF1* and *LPL* double mutants also significantly decreased LPL levels. These results are consistent with the increased severity of HTG in the double heterozygous patient, but the fact that the model simulates double homozygosity and the patient is a double heterozygote may be considered a limitation. Thus, it is reasonable to infer that this mutation affects the expression of LMF1 protein through its transcription. We could not exclude the possibility that this mutation diminishes the expression of mutant *LMF1* by reducing its stability and promoting the turnover of the protein.

The human *LMF1* gene encodes a 567 amino acid protein that contains five transmembrane domains and an evolutionarily conserved domain. This domain constitutes most of its C-terminal end, which is important for the function of LMF1 protein. The c.1523C > T mutation was located in the region recognized as being a domain of unknown function 1,222 (DUF1222). This region is found in over 50 proteins and covers a wide taxonomic range from bacteria to humans. The nonsense mutations that were recognized to be pathogenic, such as p.Tyr439Ter, p.Tyr460Ter, and p.Trp464Ter, all resulted in the large fragment deletion at the C-terminal end of the *LMF1* gene. Moreover, the activity of LPL is associated with the length of the remaining C-terminal end. Compared to patients carrying the Y439X mutation in the *LMF1* gene who lacked LPL activity, the patients carrying W464X retained 40% LPL activity. The c.1523C > T mutation in our study was also located in the C-terminal end of the LMF1 protein. This was predicted to change the tertiary structure of the protein, resulting in a defect of its function in LPL maturation. At present, the specific mechanism responsible for this is still unclear. Besides the proband, this mutation was also found in his mother who had normal TG levels. The probable explanation is that additional proteins are involved in the maturation and secretion of LPL, and partially compensate for the function of LMF1. SEL1L also plays an important role in the maturation and secretion of active LPL. In the absence of SEL1L, LPL remains and aggregates in the endoplasmic reticulum, and is then degraded through autophagy (Sha et al., 2014).

Metformin increases the expression of LMF1 in the heart, suggesting that the mechanism of metformin in reducing TG may be associated with LMF1. Contrarily, fenofibrate has no effect on the expression of LMF1 protein (Forcheron et al., 2009). The proband did not respond well to the lipid-lowering fenofibrate therapy; plasma TG levels did not return to normal. This may have resulted from the deficiency in the expression of the LMF1 protein that could not have been compensated for by fenofibrate.

Besides affecting the TG levels through LPL, the LMF1 protein also influences cholesterol concentrations in plasma. The proband’s mother, who carried the *LMF1* gene mutation, had TG values in the normal range but presented with hypercholesterolemia. The possible mechanism by which this happens is that LMF1 can regulate the redox homeostasis in the endoplasmic reticulum, thereby affecting not only the proper folding of LPL, but also the formation of disulfide bonds necessary for other secretory proteins such as fibronectin and LDLR (Roberts et al., 2018). Therefore, the decrease in LDLR levels leads to the deficiency in the ability to clear cholesterol in plasma, thereby increasing the plasma cholesterol level.

## Conclusion

In summary, A missense mutation (c.590G > A) in exon 5 of the *LPL* gene and a missense mutation (c.1523C > T) in exon 10 of the *LMF1* gene were identified in a severe case of HTG. Both the mutations reduced the mass of LPL. The combination of *LMF1* and *LPL* gene mutations significantly decreased LPL mass, which contributes to severe hypertriglyceridemia. Our study underscores the complexity of hypertriglyceridemia and the need for the combination of extensive molecular genetic testing and clinical characterization; in addition, expands the spectrum of *LMF1* mutations. Finally, it helps the family members prevent and treat the disease.

## Data Availability

The original contributions presented in the study are included in the article/Supplementary Material, further inquiries can be directed to the corresponding authors.

## Abbreviations

APOC2, apolipoprotein C2; APOA5, apolipoprotein A5; GPIHBP1, glycosylphosphatidyl-inositol-anchored high-density lipoprotein binding protein 1; HTG, hypertriglyceridemia; HEK293T cells, Human embryonic kidney 293T cells; LMF1, Lipase maturation factor 1; LPL, lipoprotein lipase; TG, triglyceride; VLDL, very-low-density lipoprotein.
